# Supplementary material for: Identification of photoperiod-induced specific miRNAs in the adrenal glands of Sunite sheep (Ovis aries)
Source: Front Vet Sci. 2022 Jul 22;9:888207. doi: 10.3389/fvets.2022.888207 (PMC9354845; doi:10.3389/fvets.2022.888207)
Supplement: Supplementary file 1 [file Data_Sheet_1.ZIP › Supplementary file/miRDeep2 file.zip!/miRDeep2 file/Novel_Structure/SP42A2/Novel_453.pdf]

Provisional ID	:	NC_019458.2_621
Score total	:	1.5
Score for star read(s)	:	-1.3
Score for read counts	:	0
Score for mfe	:	1.2
Score for randfold	:	1.6
Score for cons. seed	:	
Total read count	:	61
Mature read count	:	61
Loop read count	:	0
Star read count	:	0

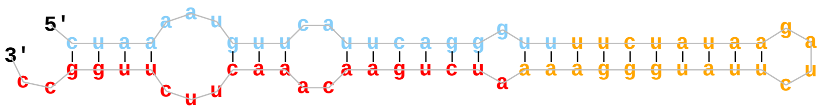

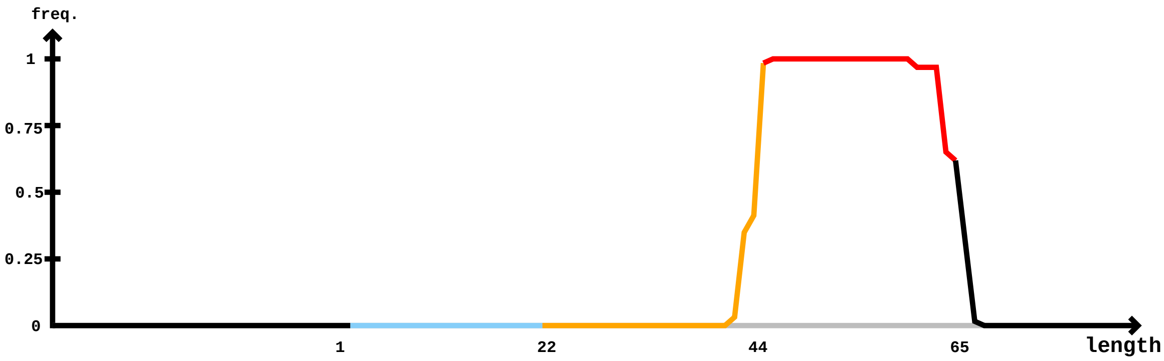

Star Mature

```
5'- uuucuguaaguucaauauuauuggguaggcuaaaauguucauucaggguuuucuauaagaucuuaugggaaaaucugaacaaacuucuuggccaacccaauccuucccaaau   -3'    exp
    ...................(((((((.((((((...(((..((((((.((((((((((....)))))))))).))))))..)))...)))))).))))))).........         reads      mm          sample
    .....................................................................aaaaucugaacaaacuuUu........................         2          1           seq
    ......................................................................aaaucugaacaaacuucuugg.....................         1          0           seq
    ......................................................................aaaucugaacaaacuuUuugg.....................         19         1           seq
    .......................................................................aaucugaacaaacuuUuuggc....................         2          1           seq
    .......................................................................aaucugaacaaacuuUuuggcc...................         2          1           seq
    ........................................................................aucugaacaaacuuUuuggcc...................         25         1           seq
    ........................................................................aucugaacaaacuuUuuggcca..................         11         1           seq
    .........................................................................ucugaacaaacuuUuuggccaa.................         1          1           seq
```
